# Postpartum depression in the Occupied Palestinian Territory: a longitudinal study in Bethlehem

**DOI:** 10.1186/s12884-016-1155-x

**Published:** 2016-11-25

**Authors:** Sara Qandil, Samah Jabr, Stefan Wagler, Simon M. Collin

**Affiliations:** 1 School of Social & Community Medicine, University of Bristol, Oakfield House, Oakfield Grove, Bristol, BS8 2BN UK; 2 Community Mental Health Center, Palestinian Ministry of Health, Ramallah, Palestine, State of; 3 The Guidance and Training Center for the Child and the Family (GTC), 153 Manger Street, Bethlehem, Palestine, State of

**Keywords:** Postpartum depression, Postnatal depression, Arabic, Palestine

## Abstract

**Background:**

Postpartum depression (PPD) affects women from different cultures around the world. No previous studies have investigated PPD among women in Palestine. Fertility rates in Palestine are among the highest in the world, hence even low rates of PPD could have considerable national impact. The aim of this study was to determine the prevalence of, and risk factors for, PPD among Palestinian mothers.

**Methods:**

101 mothers were recruited during the registration of their child’s birth (within 1 week) at the Bethlehem branch of the Ministry of Interior. Participants were assessed via a face to face interview, and were followed up 1 week, 2 weeks, 6 weeks, 3 months, and 6 months later by telephone interview. Interviews included the Arabic Edinburgh Postnatal Depression Scale (EPDS), with PPD indicated by depressive symptoms (EPDS score ≥11) at ≥2 follow-up time points. Pearson’s correlation was calculated between repeated EPDS scores, and multivariable logistic regression was used to investigate risk factors for PPD.

**Results:**

The prevalence of depressive symptoms was fairly constant (14–19%) over the follow-up period. Most depressive symptoms developed within 1 month of delivery; mothers with depressive symptoms at 3 months postpartum were highly likely to still have symptoms at 6 months. 27.7% (28/101) of women met our criteria for PPD. High parity (odds ratio (OR) 4.52 (95% CI 0.90, 22.8) parity 3+ *versus* primiparous), unplanned pregnancy (OR 2.44 (0.99, 6.01)) and sex of child not being the one desired (OR 5.07 (1.12, 22.9)) were associated with PPD, but these associations were attenuated in multivariable analysis.

**Conclusions:**

The prevalence of PPD in Palestine appears to be higher than in high income countries, but similar to the prevalence in other Middle Eastern countries. High parity and unplanned pregnancy were identified as risk factors for PPD, suggesting that fully meeting the need for family planning could reduce the incidence of PPD in the Palestinian population.

## Background

Postpartum depression (PPD) (also known as ‘postnatal depression’) is a complex and challenging depressive disorder affecting 10–15% of women worldwide [1]. This widely cited range is predominately based on studies in high income countries, and may not represent the global magnitude of the problem [2]. A higher prevalence of PPD is generally reported in women from low income countries [3], but there are large gaps in the literature, particularly in relation to Middle Eastern and Arab countries [4]. Consistently high rates of PPD (21–22%) among Arabic women have been reported in Jordan [5], the United Arab Emirates [6, 7] and Lebanon [8]. PPD prevalence among Arab-speaking populations in Israel has ranged from 8% in the north of the country [9], to 25% in a central area [10], and 26% among Bedouin populations in the south [11]. There has been no published research estimating the prevalence or nature of PPD in the occupied Palestinian territory (OPT).

Several risk factors have been identified for PPD in Arabic women, including multiparity, poor relations with partner and/or in-laws, polygamy, infant’s gender, whether or not the baby was planned, and lack of social support [12]. Although many of these factors are relevant to women globally, some are more closely related to Arabic societies, where beliefs are sometimes inextricably intertwined with Islamic values and culture [7]. Fertility rates in Palestine are notably high, at 4.4 births per woman from 2007–2010 [13]. It has been suggested that this may reflect a pattern of fertility rising in the aftermath of war [14]. Some studies among Arab women have shown no relationship between parity and PPD [8, 15, 16], whilst others have reported that higher parity is associated with low postpartum mood [12, 17]. Multiparity was thought to increase the risk of PPD by increasing the workload and responsibilities of the mother. In addition, there is an obvious financial burden associated with extra childcare [18]. Conversely, multiparity has been identified as a protective factor - in the United Arab Emirates, for example, primiparous mothers were found to report higher rates of depression [7].

PPD is known to have deleterious impacts on the mother’s personal adjustment, marital relationship and mother-infant communication [19]. If left untreated, postpartum depression can lead (in rare cases) to maternal suicide and infanticide [20]. Since Palestinian society has a high fertility rate, even a low prevalence of PPD could have considerable adverse public health impact [21]. The aim of our study was to estimate the prevalence of depressive symptoms and PPD among women in the occupied Palestinian territory, to describe the characteristics of depressive symptoms, including onset and duration, and to investigate risk factors associated with PPD. We classified women as having PPD if they experienced depressive symptoms on two or more occasions from 1 week to 6 months after birth, to differentiate PPD from ‘postpartum blues’ (which occurs in 50–85% of women, peaking around the fourth day and resolving by the tenth day following delivery) [22].

## Methods

### Design

Prospective longitudinal study using data collected in Bethlehem, OPT, solely for the purpose of the study.

### Setting

The Bethlehem Governorate covers an area of the West Bank, South of Jerusalem and has a population of approximately 216,114 people [23]. Its principal city and district capital is Bethlehem. The governorate consists of 10 municipalities, 3 refugee camps and 58 rural districts. Israeli measures including the construction of the West Bank barrier, expansion of illegal settlements, and the zoning of the majority of Bethlehem governorate as ‘Area C’ have had a crushing economic and social impact on the population. Labelling regions as ‘Area C’ means that Israel retains security control and jurisdiction over planning and construction. Since permits for Palestinian construction are rarely granted, this has reduced Bethlehem’s development space and denied residential and industrial expansion [24]. The structural and political conditions that the population endure can be recognised as infringing on key determinants of population health [25]. The Guidance and Training Center (GTC) is a mental health clinic and registered non-governmental organisation (NGO) in Bethlehem. In addition to providing mental health services in the region, the centre conducts training programs and research projects. GTC designed the present study and has full ownership of the study data.

### Procedure

Participants were recruited at the Bethlehem branch of the Ministry of Interior. By Palestinian law, newborn children have to be registered at the Ministry of Interior within a few days after birth. Parents who came to register their child’s birth were informed orally and with an information sheet about the study, and consent was sought. Consenting parents filled out a contact sheet and signed a consent form. Approximately 1 week later, a research assistant from GTC visited the mothers at their home and conducted the initial interview with them. Mothers were followed up by phone interviews at: 1 week, 2 weeks, 6 weeks, 3 months, and 6 months after the initial interview. Data were collected from October 2013 to March 2015.

### Participants

A sample of 101 mothers was obtained by identifying consecutive eligible participants presenting to register their child at the Ministry of Interior. The sample size was determined by the planned period of recruitment (18 months) and the work schedule of the researcher, rather than a priori based on the expected prevalence of PPD or effect sizes for risk factors. Inclusion criteria were: having delivered in the previous 7 days; being an Arabic-speaking Palestinian; living in the West Bank; and age 18 years or older.

### Data collection

#### Initial interview

The initial interview was modelled on a standard clinical interview, and was designed to collect sociodemographic data and information about the mother’s medical history, most recent delivery, previous births, and current and pre-pregnancy depressive symptoms. The interviewer also asked about characteristics of the newborn child and the mother’s perceived level of social support.

#### Follow-up interview

The aim of this interview was to record occurrence of depressive symptoms. Each interview ended with an open question which provided the mother with an opportunity to reveal any additional information she felt the need to share.

#### Depressive symptoms

We used the Edinburgh Postnatal Depression Scale (EPDS) to record the prevalence of depressive symptoms at the initial interview and at each follow-up time point. This 10-item scale is specifically designed to screen for PPD in community samples [26]. It produces a sum score that can range between 0 and 30. We used the Arabic version of EPDS, translated and validated by Ghubash et al. [27]. Whilst an original cut-off score of 13 and above is suggested for the English scale, it is not completely clear which is the best cut-off point for the Arabic translation. Since no validation study with Palestinian mothers exists, we chose a threshold of 11 as indicating ‘depressive symptoms’, because it was midway between the lower cut-off recommendation [28] and cut-offs used in other studies in the region (Morocco [15], Lebanon [8], Egypt [17], Jordan [5]). However, we also explored prevalence of depressive symptoms using a ‘low’ threshold of ≥9 and prevalence of ‘borderline’ depressive symptoms corresponding to an EPDS score between 9 and 10. The EPDS was administered twice at the initial interview, firstly for the mother’s current mood and thoughts and secondly asking the mother to remember how she felt the month before she learned about her pregnancy. The retrospective EPDS aimed to identify mothers who had pre-existing (‘pre-pregnancy’) depressive symptoms.

#### Postpartum depression (PPD)

We classified women as having PPD if depressive symptoms (EPDS ≥11) occurred at 2 or more follow-up time points (our own pragmatic definition to avoid spurious false positives, and to differentiate PPD from ‘postpartum blues’). PPD was used as a binary outcome in our multivariable analysis.

#### Other variables

The following variables were recorded as characteristics of the sample and potential risk factors for PPD: mother’s age; mother’s level of education; place of residency (urban, rural or refugee camp); place of delivery (home or hospital); type of delivery; planned pregnancy; parity (excluding most recent birth); sex of child; whether sex of child was as desired; and child’s birth weight. Education level was defined as ‘High School’ (secondary), ‘Tawjihi’ (International Baccalaureate level) or ‘Tertiary’ (e.g. University). High parity was defined as ≥4 previous births, except in our risk factor analysis, where we used ≥3 previous births to avoid small numbers.

### Statistical analysis

#### Phenomenological analysis

In order to investigate the prevalence and characteristics of PPD in the study population, the sample was divided into ‘PPD categories’ based on EPDS scores. Analytical categories were generated using a phenomenological method, in which qualitative content analysis is applied to non-textual data [29]. This approach was informed by inspection of the data and the opinion of a clinical psychologist in conjunction with evidence from other studies [30]. Categories were given narrative names to describe the pattern of depressive symptoms, and these were plotted over time for each category. We report the sociodemographic, obstetric and child characteristics for women in each category, and we used Kruskal-Wallis tests (for age, parity, education level), 1-way ANOVA (for birth weight), and Fisher’s exact test (for categorical variables) to test for evidence of differences in across the categories (alpha = 0.05). Pearson’s correlation coefficients were calculated between EPDS scores at different time points to assess the temporal relationship between scores. Data analysis was performed using PSPP (Free Software Foundation. 2015. GNU PSPP: Version 0.8.5. Computer Software. Boston, MA).

#### Risk factors for PPD

We compared median age and mean birth weight (for twins, the mean birth weight of the pair) in women with and without PPD using Kruskal-Wallis and Student’s *t* test, respectively (alpha = 0.05). We used logistic regression to investigate risk factors for PPD. Variables identified as risk factors in univariable analyses (*p* < 0.1) were carried forward to a multivariable analysis to obtain mutually adjusted estimates, i.e. we built a predictive model with the aim of identifying variables independently associated with the outcome (PPD) after adjustment for all other variables. Logistic regression was performed using Stata (StataCorp. 2015. Stata Statistical Software: Release 14. College Station, TX: StataCorp LP).

## Results

### Characteristics of the sample

The median age of the 101 women in the sample was 26 years (IQR 23–30 years), with a range of 18–39 years (Table 1). The majority of women reached tertiary education (degree or diploma), whilst a small proportion did not continue studying after high school. Over half of the women interviewed lived in rural areas, slightly fewer lived in cities, and a small minority resided in one of the three refugee camps in Bethlehem. Almost all of the births took place in hospital (97%), and the majority were vaginal deliveries (80%). Half of the pregnancies were unplanned (49%). One fifth of women were primiparous (18%): the median parity was 2, and the highest parity was 7 (1 woman). Among the 8 children whose sex was not the one desired, 7 were female. Two mothers gave birth to boy-girl twins, with birth weights of 2.3 kg/2.5 kg and 3.0/3.0 kg.

**Table 1:** Characteristics of the sample (*N* = 101)

	n (%)
Mother’s age (years)	
< 25	37 (37%)
25–35	55 (55%)
> 35	9 (9%)
Mother’s education	
High School	13 (13%)
Tawjihi (International Baccalaureate level)	31 (31%)
Tertiary (University or Technical/Community College)	57 (56%)
Residency	
Refugee Camp	8 (8%)
Urban	42 (42%)
Rural	51 (51%)
Place of delivery	
Home	3 (3%)
Hospital	98 (97%)
Type of delivery	
Vaginal	80 (80%)
Caesarean	21 (21%)
Planned pregnancy	
Yes	52 (52%)
No	49 (49%)
Parity	
Primiparous	18 (18%)
1	25 (25%)
2	22 (22%)
3	18 (18%)
≥ 4	18 (18%)
Sex of child^a^	
Male	48 (47%)
Female	55 (53%)
Sex of the child was the one desired	
Yes	93 (93%)
No	8 (8%)
Birth weight (kg)^a^	
< 2.5	12 (12%)
2.5–2.9	12 (12%)
3–3.49	40 (39%)
3.5–3.9	23 (22%)
≥ 4	16 (16%)

^a^ *N* = 103 children, including two pairs of boy-girl twins

### Prevalence and characteristics of depressive symptoms

Only four out of 101 women (3.96%) recalled pre-pregnancy depressive symptoms. Among women who did not have pre-pregnancy symptoms, the overall prevalence of depressive symptoms (EPDS score ≥11) was fairly constant (14–19%) over the 6 follow-up time points (Table 2), although prevalence did appear to be slightly higher between 2 and 7 weeks postpartum. This pattern was more pronounced when the threshold for depressive symptoms was relaxed (EPDS score ≥9), with highest prevalence between 2 and 7 weeks postpartum (37–40%), compared with 23% at 1 week, and 31–32% at 13–25 weeks postpartum. 47% of mothers reported postpartum depressive symptoms at least once.

**Table 2:** Mean EPDS scores and proportions of mothers with postpartum depressive symptoms (at different thresholds) at each time point (*n* = 97)^a^

Time point	EPDS score	Borderline Depressive Symptoms (EPDS score 9–10)	Depressive Symptoms (EPDS score ≥11)	Any Depressive Symptoms (EPDS score ≥9)
	Mean (SD)	% (95% CI)	% (95% CI)	% (95% CI)
1 week postpartum	6.58 (4.22)	6.2 (1.3, 11.1)	16.5 (9.0, 24.0)	22.7 (14.2, 31.2)
2 weeks postpartum	6.89 (4.67)	18.6 (10.7, 26.4)	18.6 (10.7, 26.4)	37.1 (27.3, 50.0)
3 weeks postpartum	6.90 (3.77)	20.6 (12.4, 28.8)	17.5 (9.8, 25.2)	38.1 (28.3, 50.0)
7 weeks postpartum	7.37 (3.45)	22.7 (14.2, 31.2)	17.5 (9.8, 25.2)	40.2 (30.3, 50.1)
13 weeks postpartum	7.10 (3.45)	16.5 (9.0, 24.0)	15.5 (8.1, 22.8)	32.0 (22.5, 41.4)
25 weeks postpartum	7.05 (3.28)	16.5 (9.0, 24.0)	14.4 (7.3, 21.6)	30.9 (21.6, 40.3)

^a^ Excluding four out of 101 women who reported pre-pregnancy depressive symptoms

### Phenomenological analysis

**Fig. 1:**
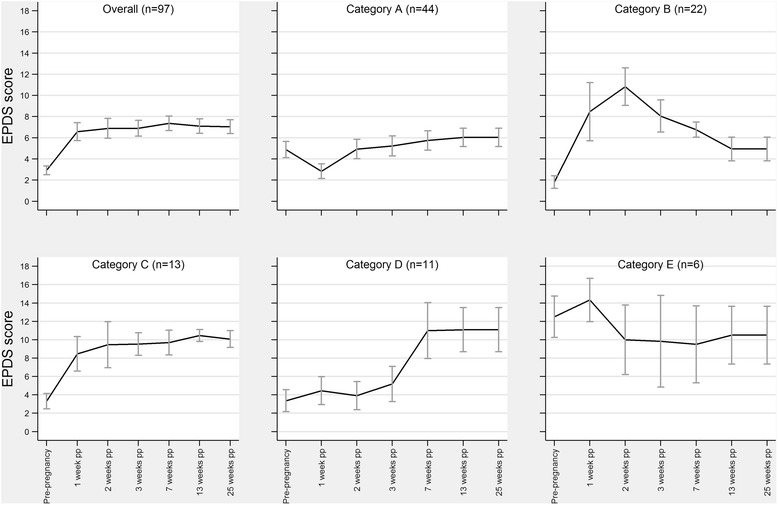
Postnatal depression categories, showing mean (95% CI) EPDS scores at each time point (see Table 3 for category definitions)

Five main categories were identified through phenomenological analysis (Table 3, Fig. 1). The categories captured variation in onset and duration of depressive symptoms. Five mothers whose scores did not appear to comply with any of the five main categories were assigned to a sixth category, labelled ‘Other’. Mothers in Category E had above-threshold or near-to-threshold scores before pregnancy, suggesting a previous occurrence of depressive symptoms. Mean EPDS scores for these women remained near-threshold or above-threshold for the duration of the follow-up period, suggesting that the heightened level of depressive symptoms occurring before pregnancy was likely to persist after childbirth. There were considerably more mothers in the early onset groups compared to late onset, suggesting that depressive symptoms were most likely to develop within the first month after delivery. Early onset depressive symptoms were likely to decline by 7 weeks post-delivery and only 37% of them persisted after this time.

**Table 3:** Postpartum depression categories defined by phenomenological analysis

Category	Category definition	Narrative name of category
C_A_	Sub-threshold^a^ scores throughout	No depressive symptoms
C_B_	Above threshold^b^ scores within the first month after delivery which decrease to subthreshold by 7 weeks postpartum	Early onset of depressive symptoms which decline over time
C_C_	Above threshold scores within the first month after delivery which do not tend to decline after 7 weeks postpartum	Early onset of depressive symptoms which persist over time
C_D_	Above threshold scores at or after 7 weeks postpartum, but lower scores before this point	Late onset depressive symptoms
C_E_	Near or above threshold^c^ scores before pregnancy	Pre-pregnancy depressive symptoms
Other	Those that didn’t fit categories C_A_ – C_E_	

^a^EPDS score <11; ^b^ EPDS score ≥11; ^c^ EPDS score ≥9

There were no differences in maternal age (*p* = 0.94), parity (*p* = 0.94), or birth weight (*p* = 0.28) between categories, but there was some evidence of variation in level of education (*p* = 0.04), with lowest levels in mothers with pre-pregnancy depressive symptoms (Table 4).

**Table 4:** Maternal, obstetric and child characteristics in each PPD category

	C_A_	C_B_	C_C_	C_D_	C_E_	Other	*p*-value^a^
	*n* = 44	*n* = 22	*n* = 13	*n* = 11	*n* = 6	*n* = 5	
Maternal age (years), median (IQR)	26 (23–30)	26 (23–34)	26 (25–34)	25 (22–35)	26 (23–26)	27 (24–30)	*p* = 0.94
Parity, median	2	1.5	2	2	2	2	*p* = 0.94
Birth weight (kg), mean (SD)	3.4 (0.6)	3.3 (0.5)	3.0 (0.7)	3.3 (0.7)	3.2 (0.7)	2.9 (0.6)	*p* = 0.28
Mother’s education level, median^b^	2	2	2	1	0.5	2	*p* = 0.04
Child's sex = male	27 (61%)	13 (59%)	6 (50%)	3 (30%)	1 (17%)	4 (80%)	*p* = 0.30
Child’s sex as desired	42 (95%)	20 (91%)	10 (77%)	11 (100%)	6 (100%)	4 (80%)	*p* = 0.18
Planned pregnancy	24 (55%)	11 (50%)	7 (54%)	6 (55%)	2 (33%)	2 (40%)	*p* = 0.94
Hospital delivery	43 (98%)	22 (100%)	13 (100%)	11 (100%)	6 (100%)	3 (60%)	*p* = 0.02
Caesarean birth	11 (25%)	3 (14%)	3 (23%)	2 (18%)	0 (0%)	2 (40%)	*p* = 0.59

^a^Kruskal-Wallis for age, parity, education level; 1-way ANOVA for birth weight; Fisher’s exact for proportions

^b^Level: 0 = High School; 1 = Tawjihi; 2 = Tertiary (e.g. University)

EPDS scores were positively correlated with each other at adjacent time points (Table 5). Scores within the first month show relatively weak correlations with each other, suggesting that occurrences of depressive symptoms during this period were more likely to be transient. In contrast, there were stronger correlations between adjacent time points in the later follow-up period (13 weeks to 25 weeks).

**Table 5:** Pearson’s Correlation Coefficients between EPDS scores at each time point (*N* = 101)

	EPDS score at 1 week	EPDS score at 2 weeks	EPDS score at 3 weeks	EPDS score at 7 weeks	EPDS score at 13 weeks	EPDS score at 25 weeks
EPDS score at 1 week	-	.30**	.20*	-.03	.05	.05
EPDS score at 2 weeks		-	.32***	.15	.05	.04
EPDS score at 3 weeks			-	.38***	.17**	.16
EPDS score at 7 weeks				-	.61***	.58***
EPDS score at 13 weeks					-	.96***
EPDS score at 25 weeks						-

* *P* ≤ 0.05; ** *P* ≤ 0.01; *** *P* ≤ 0.001 [2-tailed]

### Risk factors for PPD

PPD was reported by 27.7% (95% CI 19.3, 37.5%) of women (28/101). Women with PPD were slightly older (median 27 years, IQR 25 to 34 years) than women without PPD (median 25, IQR 23 to 29 years, *p* = 0.08). There was no difference in mean birth weight comparing babies born to women with PPD (3.19 ± 0.64 kg) with babies born to women without PPD (3.31 ± 0.61 kg, difference = -0.12 kg (95% CI -0.39 to 0.16 kg), *p* = 0.40).

PPD was reported by higher proportions of women whose pregnancy was unplanned and by women who gave birth to a child whose sex was not the one they desired (Table 6). Women whose pregnancy was unplanned had 2.4-fold higher odds of PPD (odds ratio (OR) 2.44 (0.99, 6.01), *p* = 0.05). Women who gave birth to a baby whose sex was not that desired had 5-fold higher odds of two or more PPD episodes (OR 5.07 (95% CI 1.12, 22.9), *p* = 0.04). PPD tended to be positively associated with higher parity: women with 3 or more children were 4.5 times more likely to have PPD (OR 4.52 (0.90, 22.8), *p* = 0.07), compared with primiparous women.

**Table 6:** Factors associated with postpartum depression (PPD) (*N* = 101)

Characteristics			Odds Ratio (95% CI)
	No PPD	PPD^a^	
Age (years)			
< 25	30 (81.1%)	7 (18.9%)	Ref (1.00)
25–35	37 (67.3%)	18 (32.7%)	2.08 (0.77, 5.65)
> 35	6 (66.7%)	3 (33.3%)	2.14 (0.43, 10.7)
Education level			
High School	7 (53.9%)	6 (46.2%)	Ref (1.00)
Tawjihi	21 (67.7%)	10 (32.3%)	0.56 (0.15, 2.09)
Tertiary	45 (79.0%)	12 (21.1%)	0.31 (0.09, 1.10)
Residency			
Refugee Camp	5 (62.5%)	3 (37.5%)	Ref (1.00)
Urban	32 (76.2%)	10 (23.8%)	0.52 (0.11, 2.57)
Rural	36 (70.6%)	15 (29.4%)	0.69 (0.15, 3.28)
Place of delivery			
Home	1 (33.3%)	2 (66.7%)	Ref (1.00)
Hospital	72 (73.5%)	26 (26.5%)	0.18 (0.02, 2.08)
Type of delivery			
Vaginal	58 (72.5%)	22 (27.5%)	Ref (1.00)
Caesarean section	15 (71.4%)	6 (28.6%)	1.05 (0.36, 3.06)
Planned pregnancy			
Yes	42 (80.8%)	10 (19.2%)	Ref (1.00)
No	31 (63.3%)	18 (36.7%)	2.44 (0.99, 6.01)
Parity			
Primiparous	16 (88.9%)	2 (11.1%)	Ref (1.00)
1	20 (80.0%)	5 (20.0%)	2.00 (0.34, 11.7)
2	14 (63.6%)	8 (36.4%)	4.57 (0.83, 25.2)
≥ 3	23 (63.9%)	13 (36.1%)	4.52 (0.90, 22.8)
Sex of child^b^			
Male	31 (67.4%)	15 (32.6%)	Ref (1.00)
Female	40 (75.5%)	13 (24.5%)	0.67 (0.28, 1.62)
Sex of the child was as desired			
Yes	70 (75.3%)	23 (24.7%)	Ref (1.00)
No	3 (37.5%)	5 (62.5%)	5.07 (1.12, 22.9)
Birth weight (kg)			
< 2.5	9 (75.0%)	3 (25.0%)	Ref (1.00)
2.5–2.9	5 (45.5%)	6 (54.6%)	3.60 (0.62, 21.0)
3–3.49	37 (77.1%)	11 (22.9%)	0.89 (0.21, 3.88)
3.5–3.9	9 (64.3%)	5 (35.7%)	1.67 (0.30, 9.16)
≥ 4	13 (81.3%)	3 (18.8%)	0.69 (0.11, 4.24)

^a^ Depressive symptoms (EPDS ≥11) on ≥2 occasions; ^b^ Excluding two mothers who gave birth to boy-girl twins

Parity, planned pregnancy and desired sex of child were carried forward to a multivariable model. The association of high parity with PPD was much less evident when adjusted for the other two variables (OR 3.40 (0.65, 17.8), *p* = 0.15, parity 3+ vs primiparous). The associations of unplanned pregnancy (OR 2.34 (0.90, 6.06), *p* = 0.08) and sex of child not being the one desired (OR 4.65 (0.95, 22.7), *p* = 0.06) were also attenuated. Although statistical evidence for these adjusted associations was weak, the ORs are only slightly changed. This multivariable analysis was not adjusted for age, since this was very strongly correlated with parity (correlation coefficient = 0.73, P ≤ 0.001).

## Discussion

Our study provides further evidence that postpartum depression is as much a maternal health issue in Arab and Middle Eastern countries as it is in the West. Using criteria of an EPDS score ≥11 on ≥2 occasions, we found a PPD prevalence of 28%. This is higher than the 10–15% prevalence reported for women worldwide [1], but consistent with the 20–30% prevalence found in other Arabic communities [5–8, 11]. Our results showed Palestinian mothers developing depressive symptoms that were not reported before pregnancy.

It is possible that the onset and duration of PPD is influenced by cultural practices. PPD as defined in DSM-V has onset during pregnancy or within 4 weeks of delivery [31], and cohort studies in high-income countries report typical onset between the second trimester [32] and the first week postpartum [33]. In contrast, a recent study in Egypt reported onset of depressive symptoms as occurring most frequently between the second and third months postpartum, and persisting for >6 months [17]. This late onset of depressive symptoms in Arabic societies has been ascribed to a popular cultural belief that the puerperium is a period of uncleanliness, prohibiting women from performing their normal household chores [17]. Instead, the mother, assisted by her family, is allowed to recuperate for 40 days after childbirth [4]. The support provided within this period may delay the onset of major depression and ameliorate mood disturbances until after the first postnatal month. However, in our cohort, depressive symptoms developed most frequently within 1 month postpartum, with only one fifth of depressive symptoms being classified as ‘late onset’.

With regard to duration, approximately two thirds of women who experienced depressive symptoms within the first month postpartum did not report symptoms 1 month later, with the remaining third continuing to experience symptoms for the duration of the study. There was strong correlation between EPDS scores at 3 and 6 months, meaning that mothers in our study who were depressed at 3 months were still likely to be depressed 3 months later. These mixed patterns of declining early postpartum symptoms for the majority and persistent postpartum symptoms in a substantial minority have been observed in other studies [30]. Similarly, the small number of mothers in our study who experienced pre-pregnancy depressive symptoms also had symptoms throughout the follow-up period, which is consistent with a previous history of depression being a risk factor for a chronic course of PPD [30]. Although our retrospective use of the EPDS has not been validated, the relationship between previous depression and PPD has been robust in different study designs [34].

Our results showed a trend for women with ≥3 children to have greater odds of developing PPD, which is consistent with systematic reviews that have identified high parity as a risk factor for PPD [35, 36]. That women with unplanned pregnancies showed a trend of increased risk of PPD replicates findings from an earlier study among Palestinian women [37], and is consistent with two other studies in Arabic populations [5, 17]. Saleh et al. highlighted the difficulties encountered by Arab women in adjusting to an unplanned pregnancy, which could plausibly trigger PPD [17]. The high level of unplanned pregnancies among women in our study (49%) suggests a role for provision of family planning in reducing the incidence of PPD in this population. Although unmet need for family planning in Bethlehem from 2007–2010 was relatively low at 8.3%, in other parts of Palestine it was as high as 22% [13].

Patriarchal traditions and gender prejudices rooted within Palestinian culture are related to a preference for male children [38], and studies from countries where gender inequality is deeply entrenched have suggested that disappointment with the sex of the baby (particularly if the baby is a girl) is associated with PPD [12, 17]. These findings are consistent with our results which indicated that depression was more likely to occur when the sex of the child was not the one desired.

A major strength of our study was its longitudinal design, in which there were no losses to follow-up. Collecting data from multiple intervals allowed a precise identification of onset and duration.

The main limitation of our study is its relatively small size, which means that our estimates for prevalence and effects of risk factors have wide confidence intervals. The sample size was determined by the period of recruitment (which itself was determined by the resources available to conduct the study), and we did not have prior estimates of PPD prevalence in the population, or effect sizes for the risk factors, to calculate a minimum sample size. The sample that we obtained was selected sequentially, rather than strictly at random. Although we have no reason to suspect selection bias (because all parents are legally obliged to register their child’s birth at a single location), we do not have demographic information about women who were not recruited in order to assess whether our sample was truly representative. However, the proportion of women in our sample who had tertiary education is consistent with UNESCO statistics which show >50% of Palestinian women enrolled in tertiary education since 2007 (58% in 2011) [39], and the age distribution in our sample reflects age-specific fertility rates based on household survey data for the West Bank [40]. It is possible that other unmeasured factors might have predicted risk of PPD in this population. We did not record gestational age, but we found no association of birth weight with PND, and we would expect birth weight to be strongly correlated with gestational age.

Classifying depressive symptoms into taxonomies through phenomenological analysis was a means of illustrating that women with PPD in our study could not be considered a homogenous group, and the differences that we identified between PPD categories might be useful for future research in other populations. There is an increasing tendency in the literature towards differentiating PPD into distinct subtypes linked to specific features of onset and duration [30]. The phenomenological method utilised to generate these categories benefits from being inductive, but can be criticised for being subjective compared with, for example, a latent class analysis. We must also acknowledge that the EPDS is not a diagnostic tool, and PPD in our study is subject to misclassification. Our assessment of depressive symptoms was made only in the postpartum period, and the retrospective EPDS score may have been an unreliable measure of depression during pregnancy because depressed mothers may have been more inclined to recall low mood.

The Separation Wall and policies designed to fragment the Palestinian population have resulted in populations of Palestinian minorities that exhibit different socioeconomic circumstances and living conditions. Inevitably, such variations influence the standard of health that is attainable in each district. For example, unmet need for contraception ranges from 8.3% in Bethlehem to 22.1% in Hebron. Hence, our findings may not be generalizable to other Palestinian communities. Our findings need to be replicated in other Palestinian communities using larger samples and an EPDS cut off score that has been validated for Palestinian mothers. Ideally, data collection would start before or during pregnancy, and postpartum follow-up periods would be longer than 6 months. With regard to clinical implications, this study suggests that high parity and unplanned pregnancies, if prevented with effective family planning programs, might reduce the prevalence of PPD in this population. Future studies should investigate unmet family planning as a risk factor for PPD*.*

## Conclusions

This study found that the prevalence of PPD in Palestine (28%) was higher than in high income countries, but consistent with findings from other Middle Eastern countries. High parity and unplanned pregnancy were identified as potential risk factors for PPD. The high fertility rate and percentage of unplanned pregnancies in our study population suggests that fully meeting the need for family planning could reduce the incidence of PPD in the Palestinian population. An understanding of PPD in specific settings is important because it can help practitioners to provide better care a local level. More generally, it contributes to our understanding of the mental health needs of mothers from different cultural backgrounds.

## Abbreviations

EPDS: Edinburgh Postnatal Depression Scale
GTC: Guidance and Training Center
IQR: Interquartile range
OPT: Occupied Palestinian Territory
PPD: Postpartum Depression

## References

[1] Villegas L, McKay K, Dennis CL, Ross LE (2011). Postpartum depression among rural women from developed and developing countries: a systematic review. J Rural Health.

[2] Halbreich U, Karkun S (2006). Cross-cultural and social diversity of prevalence of postpartum depression and depressive symptoms. J Affect Disord.

[3] Howard LM, Molyneaux E, Dennis CL, Rochat T, Stein A, Milgrom J (2014). Non-psychotic mental disorders in the perinatal period. Lancet.

[4] Nahas V, Amasheh N (1999). Culture care meanings and experiences of postpartum depression among Jordanian Australian women: a transcultural study. J Transcult Nurs.

[5] Mohammad KI, Gamble J, Creedy DK (2011). Prevalence and factors associated with the development of antenatal and postnatal depression among Jordanian women. Midwifery.

[6] Ghubash R, Abou-Saleh MT (1997). Postpartum psychiatric illness in Arab culture: prevalence and psychosocial correlates. Br J Psychiatry.

[7] Green K, Broome H, Mirabella J (2006). Postnatal depression among mothers in the United Arab Emirates: socio-cultural and physical factors. Psychol Health Med.

[8] Chaaya M, Campbell OM, El Kak F, Shaar D, Harb H, Kaddour A (2002). Postpartum depression: prevalence and determinants in Lebanon. Arch Womens Ment Health.

[9] Glasser S, Tanous M, Shihab S, Goldman N, Ziv A, Kaplan G (2012). Perinatal depressive symptoms among Arab women in northern Israel. Matern Child Health J.

[10] Eilat-Tsanani S, Merom A, Romano S, Reshef A, Lavi I, Tabenkin H (2006). The effect of postpartum depression on women's consultations with physicians. Isr Med Assoc J.

[11] Glasser S, Stoski E, Kneler V, Magnezi R (2011). Postpartum depression among Israeli Bedouin women. Arch Womens Ment Health.

[12] Ramasubramaniam S, Madhavanprabhakaran G, Renganathan L, Raman S (2014). Prevalence of postnatal depression among Arab women: a narrative review. J Res Nurs Midwifery.

[13] Palestinian Central Bureau of Statistics: Final Report of the Palestinian Family Survey 2010. In. Ramallah, Palestine: Palestinian Central Bureau of Statistics; 2013.

[14] Khawaja M (2000). The recent rise in Palestinian fertility: Permanent or transient?. Popul Stud-a J Demogr.

[15] Agoub M, Moussaoui D, Battas O (2005). Prevalence of postpartum depression in a Moroccan sample. Arch Womens Ment Health.

[16] Alfayumi-Zeadna S, Kaufman-Shriqui V, Zeadna A, Lauden A, Shoham-Vardi I (2015). The association between sociodemographic characteristics and postpartum depression symptoms among Arab-Bedouin women in Southern Israel. Depress Anxiety.

[17] Saleh ES, El-Bahei W, del El-Hadidy MA, Zayed A (2013). Predictors of postpartum depression in a sample of Egyptian women. Neuropsychiatr Dis Treat.

[18] Al Hinai FI, Al Hinai SS (2014). Prospective study on prevalence and risk factors of postpartum depression in Al-dakhliya governorate in oman. Oman Med J.

[19] Josefsson A, Berg G, Nordin C, Sydsjo G (2001). Prevalence of depressive symptoms in late pregnancy and postpartum. Acta Obstet Gynecol Scand.

[20] Kaminsky LM, Carlo J, Muench MV, Nath C, Harrigan JT, Canterino J (2008). Screening for postpartum depression with the Edinburgh Postnatal Depression Scale in an indigent population: does a directed interview improve detection rates compared with the standard self-completed questionnaire?. J Matern Fetal Neonatal Med.

[21] Rahim HF, Wick L, Halileh S, Hassan-Bitar S, Chekir H, Watt G, Khawaja M (2009). Maternal and child health in the occupied Palestinian territory. Lancet.

[22] Norhayati MN, Hazlina NH, Asrenee AR, Emilin WM (2015). Magnitude and risk factors for postpartum symptoms: a literature review. J Affect Disord.

[23] Palestinian Central Bureau of Statistics: Localities in Bethlehem Governorate by Type of Locality and Population Estimates, 2007–2016. In. Ramallah, Palestine: Palestinian Central Bureau of Statistics; 2016.

[24] UN (2009). Shrinking Space: Urban Contraction and Rural Fragmentation in the Bethlehem Governorate.

[25] Giacaman R, Khatib R, Shabaneh L, Ramlawi A, Sabri B, Sabatinelli G, Khawaja M, Laurance T (2009). Health in the Occupied Palestinian Territory 1 Health status and health services in the occupied Palestinian territory. Lancet.

[26] Cox JL, Holden JM, Sagovsky R (1987). Detection of postnatal depression. Development of the 10-item Edinburgh Postnatal Depression Scale. Br J Psychiatry.

[27] Ghubash R, Abou-Saleh MT, Daradkeh TK (1997). The validity of the Arabic Edinburgh Postnatal Depression Scale. Soc Psychiatry Psychiatr Epidemiol.

[28] Group GoWASPMHR: Edinburgh Postnatal Depression Scale (EPDS): Translated versions – validated. In*.* Perth, Western Australia; 2006.

[29] Mayring P: Qualitative content analysis: theoretical foundation, basic procedures and software solution. 143 2014.

[30] Vliegen N, Casalin S, Luyten P (2014). The course of postpartum depression: a review of longitudinal studies. Harv Rev Psychiatry.

[31] American Psychiatric A, American Psychiatric A, Force DSMT (2013). Diagnostic and statistical manual of mental disorders : DSM-5.

[32] Evans J, Heron J, Francomb H, Oke S, Golding J (2001). Cohort study of depressed mood during pregnancy and after childbirth. BMJ.

[33] Hannah P, Adams D, Lee A, Glover V, Sandler M (1992). Links between early post-partum mood and post-natal depression. Br J Psychiatry.

[34] Riecher-Rossler A, Hofecker Fallahpour M (2003). Postpartum depression: do we still need this diagnostic term?. Acta Psychiatr Scand Suppl.

[35] Fisher J, Cabral de Mello M, Patel V, Rahman A, Tran T, Holton S, Holmes W (2012). Prevalence and determinants of common perinatal mental disorders in women in low- and lower-middle-income countries: a systematic review. Bull World Health Organ.

[36] Sawyer A, Ayers S, Smith H (2010). Pre- and postnatal psychological wellbeing in Africa: a systematic review. J Affect Disord.

[37] Hammoudeh W, Mataria A, Wick L, Giacaman R (2009). In search of health: quality of life among postpartum Palestinian women. Expert Rev Pharmacoecon Outcomes Res.

[38] Palestinian Central Bureau of Statistics: Palestinian Family Health Survey, 2006: Final Report. In*.* Ramallah, Palestine; 2007.

[39] Country Profiles (Palestine) [http://www.uis.unesco.org/]. Accessed 19th Sept 2016.

[40] Khawaja M: The Fertility of Palestinian Women in Gaza, the West Bank, Jordan and Lebanon. Population (English Edition, 2002-) 2003, 58(3):273.

